# Caring for A- and B-scans

**Published:** 2015

**Authors:** Ismael Cordero

**Affiliations:** Clinical Engineer, Philadelphia, USA. **ismaelcordero@me.com**

**Figure:**
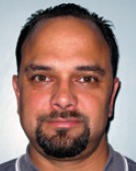
Ismael Cordero

An eye ultrasound is a test that uses high-frequency sound waves to measure and create detailed images of a patient's eye.

Ophthalmic ultrasound units (Figure 1) consist of a console, a foot pedal, one or two types of probes, and a keyboard (usually but not always). The probes contain piezo-electric crystals, which convert electrical energy into ultrasonic soundwaves in the frequency range of 8–80 MHz. These waves are sent to the tissue being examined and some of the waves are reflected, as echoes, back to the probe. These echoes are converted into electrical signals, which are processed to measure and create an image of the tissue.

There are two main types of ultrasonic scans. The B-scan probe is larger than the A-scan probe since it houses a small motor that sweeps the crystals back and forth to scan the eye.

- **A-scan** (Figure 2). This measures the length of the eye to determine the correct power of a lens implant before cataract surgery. After the patient's eye is numbed with anaesthetic drops, the small A-scan probe is placed against the cornea to make the measurements.
- **B-scan** (Figure 3). This scan provides information about the inside of the eye, usually when a patient has cataracts or other conditions that make it hard to see into the back of the eye. The B-scan probe is gently placed against the eyelids and the patient is asked to look in many different directions.

## Optimal care and use

- Properly store the probes when not in use. Most machines have a designated storage place for probes, such as a dedicated holder.
- Avoid dropping the probe or subjecting it to any kind of impact.
- Always inspect the probe, including its lens and cable, before each use.
- Do **not** use damaged probes. Injury to the operator or patient may occur! This is because cleaning and/or gel solutions may leak into the transducer, resulting in electrical shock.
- Avoid sharply bending, twisting, kinking, or pinching the cable. Excessive bending or stress on the cable may result in damage to its casing, causing an electrical shock to the patient or operator.
- Place the patient close enough to the console to avoid stretching and damaging the probe's cord.

**Figure 1.**
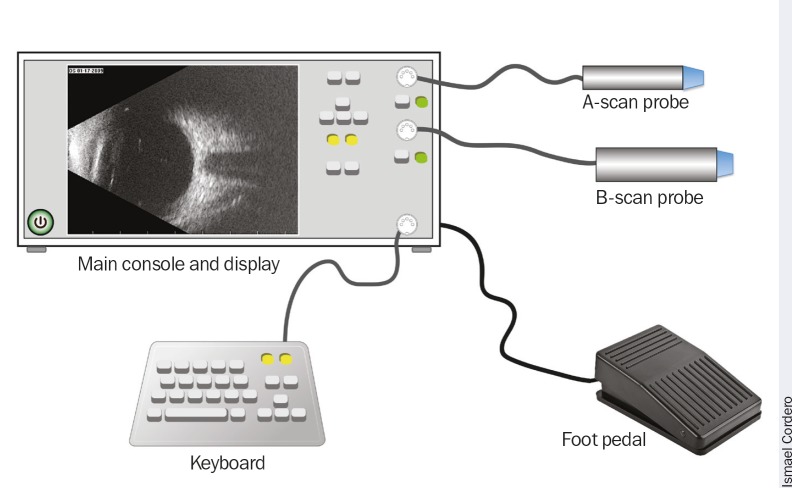


## Cleaning

- Use only approved gels and germicides and strictly follow the instructions when applying, cleaning and disinfecting transducers.
- Do **not** steam, heat autoclave, or use ethylene oxide (EO) gas processes on probes.
- Disconnect the probe from the ultrasound console and rinse the probe with a warm, non-abrasive soap and water solution.
- Do **not** immerse the probe connector.
- Meticulously scrub the probe as needed with a soft brush, sponge, or gauze pad to remove all residues.
- Air dry or dry with soft cloth or gauze pad.

## Calibration

Many ocular ultrasound units have a plastic test block that can be used as a reference for calibration verification. Units have a special mode to allow measurement of the test block; this should yield a specified measurement (for instance, axial length of 24.10 mm ± 0.25 mm). You should routinely use this block and calibration mode to test the accuracy of the unit.

**Figure 2.**
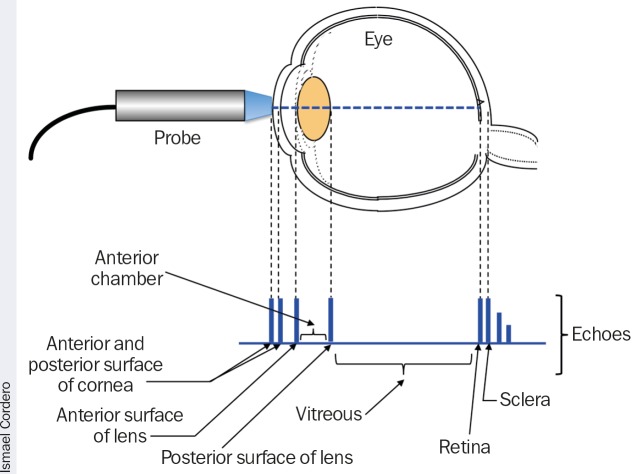
A-scan

**Figure 3.**
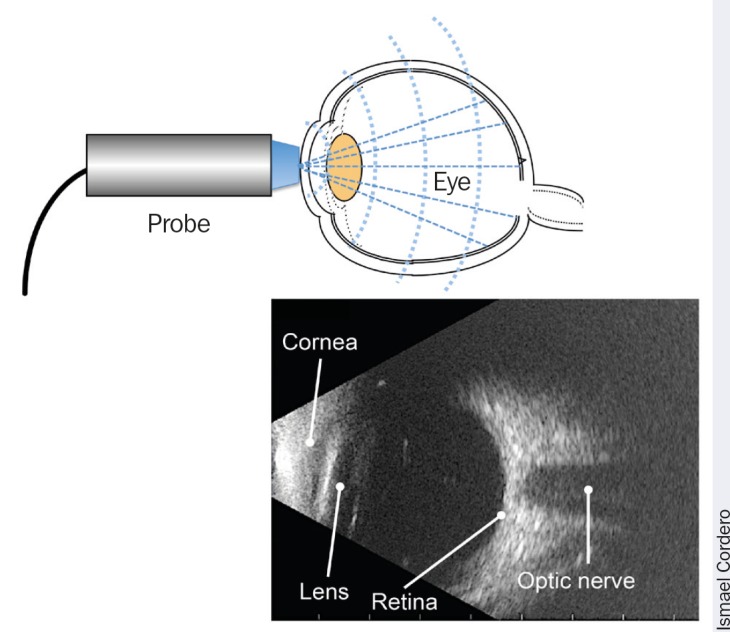
B-scan

